# Chemogenetic stimulation of the G_i_ pathway in astrocytes suppresses neuroinflammation

**DOI:** 10.1002/prp2.822

**Published:** 2021-10-22

**Authors:** Jae‐Hong Kim, Md Habibur Rahman, Won Ha Lee, Kyoungho Suk

**Affiliations:** ^1^ Department of Pharmacology School of Medicine Kyungpook National University Daegu Republic of Korea; ^2^ BK21 Plus KNU Biomedical Convergence Program Department of Biomedical Sciences School of Medicine Kyungpook National University Daegu Republic of Korea; ^3^ Brain Science & Engineering Institute Kyungpook National University Daegu Republic of Korea; ^4^ School of Life Sciences Brain Korea 21 Plus KNU Creative BioResearch Group Kyungpook National University Daegu Republic of Korea

**Keywords:** astrocyte, chemogenetics, G_i_‐DREADD, hM4Di, neuroinflammation

## Abstract

Engineered G protein‐coupled receptors (GPCRs) are commonly used in chemogenetics as designer receptors exclusively activated by designer drugs (DREADDs). Although several GPCRs have been studied in astrocytes using a chemogenetic approach, the functional role of the astrocytic G_i_ pathway is not clear, as the literature is conflicting depending on the brain regions or behaviors investigated. In this study, we evaluated the role of the astrocytic G_i_ pathway in neuroinflammation using a G_i_‐coupled DREADD (hM4Di). G_i_‐DREADD was expressed in hippocampal astrocytes of a lipopolysaccharide (LPS)‐induced neuroinflammation mouse model using adeno‐associated viruses. We found that astrocyte G_i_‐DREADD stimulation using clozapine N‐oxide (CNO) inhibits neuroinflammation, as characterized by decreased levels of proinflammatory cytokines, glial activation, and cognitive impairment in mice. Subsequent experiments using primary astrocyte cultures revealed that G_i_‐DREADD stimulation significantly downregulated LPS‐induced expression of *Nos2* mRNA and nitric oxide production. Similarly, in vitro calcium imaging showed that activation of the astrocytic G_i_ pathway attenuated intracellular calcium transients triggered by LPS treatment, suggesting a positive correlation between enhanced calcium transients and the inflammatory phenotype of astrocytes observed in the inflamed brain. Taken together, our results indicate that the astrocytic G_i_ pathway plays an inhibitory role in neuroinflammation, providing an opportunity to identify potential cellular and molecular targets to control neuroinflammation.

**Abbreviations**
ACC: Anterior cingulate cortex
CEBP: CCAAT/enhancer‐binding protein
CNS: Central nervous system
CREB: cAMP response element‐binding
DMEM: Dulbecco's modified Eagle's medium
DREADD: Designer receptors exclusively activated by designer drugs
ER: Endoplasmic reticulum
GPCR: G protein‐coupled receptors
STAT: Signal transducer and activator of transcription

## 1 INTRODUCTION

Designer receptors exclusively activated by designer drugs (DREADDs) are genetically modified G‐protein‐coupled receptors (GPCRs). DREADDs are used in chemogenetic approaches, which allow researchers to remotely control cellular activity via modulation of GPCR (G_i_, G_q_, or G_s_)‐signaling pathways with the application of selective ligands, such as clozapine N‐oxide (CNO).^1^ This strategy has commonly been used to regulate the activity of various types of neurons to study brain functions and behaviors. Recent studies have also used this technique to study glial cells, including astrocytes,^2^, ^3^ the most abundant glial cell type in the central nervous system (CNS), exhibiting notable heterogeneity in their morphology and function.^4^ Among the GPCRs in astrocytes studied using DREADDs, there are inconsistencies reported in the functional role of G_i_ signaling, which requires further exploration.

The functional role of the G_i_ signaling pathway in astrocytes has been investigated in several studies using chemogenetic approaches. A study by Nam et al. revealed that chemogenetic activation of astrocyte‐specific G_i_‐DREADD hM4Di enhanced synaptic transmission and plasticity of Schaffer collaterals in the hippocampus, thereby inducing the formation of contextual memory for conditioned place preference.^5^ Conversely, another recent study demonstrated that activation of G_i_‐coupled designer receptor hM4Di in hippocampal cornu ammonis (CA1) astrocytes during learning impairs remote, but not recent memory recall, and decreases the activity of CA1 neurons projecting to the anterior cingulate cortex (ACC) during memory retrieval.^6^ Similarly, another study found that activation of astroglial G_i_ signaling in the hippocampus was sufficient to protect against the development of stress‐enhanced fear learning, post‐traumatic stress disorder‐like behavior.^7^ Moreover, it has been demonstrated that striatal astrocyte G_i_ pathway activation corrects behavioral phenotypes in a Huntington's disease mouse model.^8^ However, the same research group previously reported that activation of an astrocyte‐specific G_i_ pathway in the striatum produced inattentive hyperactivity in mice under physiological conditions.^9^ Therefore, given the multiple contradicting results, the functional role of the astrocytic G_i_ pathway remains unclarified in both healthy and disease states. It is well accepted that in disease conditions astrocytes can undergo morphological and functional remodeling into “reactive astrocyte,” called “astrogliosis,” where normal homeostatic mechanisms are lost and proinflammatory responses occur at higher levels, contributing to neuroinflammation and associated diseases.^10^, ^11^ Astrocyte‐mediated neuroinflammation is associated with neurodegenerative and metabolic diseases, such as Alzheimer's disease (AD),^12^ Parkinson's disease (PD),^13^ traumatic brain injury (TBI),^14^ multiple sclerosis (MS),^15^ diabetes, and obesity.^16^ However, to the best of our knowledge, the role of the astrocytic G_i_ pathway in neuroinflammation has not been yet studied.

To explore the role of astrocytic G_i_ pathway in neuroinflammation, we used the designer receptor hM4Di to manipulate the G_i_ pathway in these cells in a lipopolysaccharide (LPS)‐induced neuroinflammation model. We found that chemogenetic activation of astrocytic G_i_ signaling in the hippocampus attenuates LPS‐induced neuroinflammation, as evidenced by decreased levels of inflammatory mediators, gliosis, and cognitive impairment in mice. In vitro studies using cultured astrocytes revealed that G_i_ activation in astrocytes reduced LPS‐induced expression of *Nos2* mRNA, nitric oxide (NO) production, and intracellular calcium (Ca^2+^) levels. These findings provide evidence for the important role of astrocytic G_i_ activation and downstream signaling pathways in mitigating neuroinflammation.

## 2 MATERIALS AND METHODS

### 2.1 Animals

Male C57BL/6 mice (age 8–12 weeks) were obtained from Samtaco. Only male mice were used in this study. All animal experiments were performed according to approved animal protocols and guidelines established by the Animal Care Committee of Kyungpook National University (No. KNU 2019‐09).

### 2.2 Viral gene transfer and chemogenetic stimulation in vivo

The following viral constructs were used: AAV5‐GFAP‐hM3Dq‐mCherry (VVF Zurich; viral titer 3.9 × 10^12^), AAV5‐GFAP‐hM4Di‐mCherry (VVF Zurich; viral titer 4.7 × 10^12^), and AAV5‐GFAP‐eYFP (control vector; VVF Zurich; viral titer 3.9 × 10^12^). To prepare animals for in vivo experiments, mice were anesthetized using 2%–4% isoflurane (Baxter) in oxygen and placed in a stereotaxic apparatus. For chemogenetic stimulation in vivo, two stainless steel injection needles were bilaterally injected. The needle tip was gently lowered to 0.5 mm above the hippocampus (from the bregma: 2 mm posterior, 1.8 mm lateral, and 1.2 mm dorsoventral) to limit damage to the target region. A volume of 0.5 μl of the virus at 0.1 μl/min was injected into the hippocampus bilaterally. After injection, the needle tip was held in place for 10 min before retraction to prevent leakage, and then removed. Immediate postoperative care was provided, and the animals were allowed to recover for 14 days before the experiment, to ensure high levels of transgene expression. Prior to behavioral experiments following viral gene transfer, the expression of relevant proteins within the CA1 region was confirmed via fluorescence.

### 2.3 CNO administration

CNO (Tocris Bioscience, Catalog number: 4936) was dissolved in DMSO and then diluted in 0.9% saline to yield a final DMSO concentration of 0.5%. The saline solution used for control injections also consisted of 0.5% DMSO. Before conducting the behavioral assays, 1 or 3 mg/kg CNO was intraperitoneally (i.p.) injected at 8‐h intervals for 2 days. Despite the short CNO half‐life in mouse plasma (<2 h),^17^ acute treatment of DREADD‐expressing experimental animals usually have much longer biological effects (6–10 h).^17^, ^18^, ^19^ To investigate the effect of astrocyte chronic activation, we injected CNO (1 or 3 mg/kg, i.p.) into mice at 8‐h intervals. We chose this 8‐h duration based on a previous report.^19^ For in vitro studies, primary astrocytes expressing hM3Dq‐ or hM4Di‐mCherry were treated with CNO (10 μM).

### 2.4 Intracerebroventricular injection of LPS

Under isoflurane anesthesia, mice were mounted onto a stereotaxic frame. Two guide cannulas were surgically implanted bilaterally 0.5 mm above the lateral ventricle of the brain. The coordinates for the placement of the guide cannula were as follows: 1 mm posterior to the bregma, 1.6 mm lateral, and 2.0 mm below the skull surface at the point of entry. Mice were allowed to recover for a minimum of 14 days before treatment and initiation of behavioral testing. After recovery, intracerebroventricular (i.c.v.) injections of sterile saline or lipopolysaccharide (LPS, 1 mg/ml; Sigma‐Aldrich) were conducted. CNO (1‐ or 3‐mg/kg, i.p.) injection was initiated 4 h prior to LPS injection (2 μl, i.c.v., flow rate of 0.5 μl/min).

### 2.5 Immunohistochemistry

Animals were anesthetized using diethyl ether, and transcardially perfused first with saline and then with 4% paraformaldehyde diluted in 100 mM PBS. The brains were dissected, fixed in 4% paraformaldehyde for 3 days, and then cryoprotected using a 30% sucrose solution for an additional 3 days. The fixed brains were embedded in OCT compound (Tissue‐Tek, Sakura Finetek) and then sectioned into 20‐μm‐thick slices. For immunofluorescence analysis, tissue sections were incubated with rabbit anti‐GFAP (Dako, Catalog number: Z0334), mouse anti‐NeuN (Sigma‐Aldrich, Catalog number: MAB377), and goat anti‐Iba‐1 (Novus Biologicals, Catalog number: NB100‐1028) antibodies. Sections were visualized by incubation with Cy3‐, Cy5‐, and FITC‐conjugated anti‐rabbit, anti‐mouse, or anti‐goat IgG antibody (Jackson ImmunoResearch; Cy3‐goat, Catalog number 705‐165‐147; Cy5‐rabbit, Catalog number 711‐175‐152; FITC‐mouse, Catalog number 715‐095‐151; FITC‐goat, Catalog number 705‐095‐147) and examined under a fluorescence or confocal microscope. We took the images with a Lionheart FX Automated Microscope at 10× magnification and equalized their brightness and contrast. We measured fluorescence intensity using ImageJ software version 1.44 (National Institutes of Health). To count the GFAP‐ or Iba‐1‐positive cells, we set a FITC, Cy3, or Cy5 secondary mask within the DAPI primary mask and counted the cells with co‐localized staining using Gen 5 software (BioTek).

### 2.6 Reverse transcription polymerase chain reaction (RT‐PCR)

Total RNA was extracted from hippocampal tissues and cells using the QIAzol reagent (QIAGEN) according to the manufacturer's protocol. For conventional RT‐PCR, reverse transcription was conducted using Superscript II (Invitrogen) and oligo(dT) primers. PCR amplification using specific primer sets was carried out at an annealing temperature of 55–60℃ for 20–30 cycles. PCR was performed using a DNA Engine Tetrad Peltier Thermal Cycler (MJ Research). For analysis of PCR products, 10 μl of each PCR was electrophoresed on 1% agarose gel and detected under ultraviolet light. *Gapdh* was used as an internal control. Quantitative real‐time PCR (qPCR) was performed using the one‐step SYBR^®^ PrimeScript™ RT‐PCR kit (Perfect Real‐Time; Takara Bio Inc.) according to the manufacturer's instructions, followed by detection using the ABI Prism^®^ 7000 sequence detection system (Applied Biosystems). The relative changes in gene expression determined by qPCR experiments were calculated using the 2^−ΔΔCT^ method.^20^ The primers used in qPCR analyses of mouse *Lcn2, Il1b, Tnfa, Nos2*, and *Gapdh* were as follows: *Lcn2*, 5′‐ATG TCA CCT CCA TCC TGG TC‐3′ (forward), 5′‐CAC ACT CAC CAC CCA TTC AG‐3′ (reverse); *Il1b*, 5′‐AGT TGC CTT CTT GGG ACT GA‐3′ (forward), 5′‐TCC ACG ATT TCC CAG AGA AC‐3′ (reverse); *Tnfa*, 5′‐CAT CTT CTC AAA ATT CGA GTG ACA A‐3′ (forward), 5′‐ACT TGG GCA GAT TGA CCT CAG‐3′ (reverse); *Nos2*, 5′‐GCC ACC AAC AAT GGC AAC A‐3′ (forward), 5′‐CGT ACC GGA TGA GCT GTG AAT T‐3′ (reverse); and *Gapdh*, 5′‐TGG GCT ACA CTH AHC ACC AG‐3′ (forward), 5′‐GGG TGT CGC TGT TGA AGT CA‐3′ (reverse).

### 2.7 Passive avoidance test

This test began with training, in which a mouse was placed in a light chamber; when the mouse crossed over to the dark chamber, it received a mild electric shock on the foot (0.25 mA for 1 s). The initial latency to enter the dark (shock) compartment was used as the baseline measure. During the probe trials, 24 h after training, the mouse was again placed in the light compartment, and the latency to return to the dark compartment was measured as an index of passive fear avoidance. The passive avoidance test method used for cognitive behavior assessment has a 200 s maximum latency, as observed with most of the vehicle and CNO‐treated animals. Thus, it is not suited for cognitive enhancement observation, which is similar to previously published studies.^21^ Future studies will need to address this issue using other methods such as novel object recognition task, Barnes maze, or Morris water maze tests.^22^, ^23^

### 2.8 Primary astrocyte cultures and virus infection

The brains of 3‐day‐old mice were homogenized and mechanically disrupted using a nylon mesh. The obtained mixed glial cells were seeded in culture flasks, and cultured at 37℃ in a 5% CO_2_ incubator in Dulbecco's modified Eagle's medium (DMEM) supplemented with 10% fetal bovine serum (FBS), 100 U/ml penicillin, and 100 μg/ml streptomycin. Culture media were changed initially after 5 days, and then changed every 3 days. After 14 days of culture, primary astrocytes were obtained from mixed glial cells using a mechanical shaker (200 rpm for 12 h). Then, primary astrocytes in 12‐well plates were infected with hM3Dq‐ or hM4Di‐mCherry virus. After 7 days of virus infection, the cells were used for experiments.

### 2.9 Nitrite quantification

Astrocyte cultures were treated with stimuli in 96‐well plates, and then NO_2_^−^ in culture media was measured in order to assess NO production levels by the Griess reaction as described previously.^24^ Sample aliquots of 50 μl were mixed with 50 μl of Griess reagent (1% sulfanilamide, 0.1% naphthylmethyl diamine dihydrochloride, 2% phosphoric acid) in 96‐well plates, and then incubated at 25℃ for 10 min. The absorbance at 540 nm was measured using a microplate reader (Anthos Labtec Instruments). NaNO_2_ was used as a standard to calculate NO_2_ concentration.

### 2.10 Intracellular Ca^2+^ measurement

To measure intracellular Ca^2+^ in cultured astrocytes, we grew primary astrocytes on glass coverslips and washed them three times with a solution containing 150 mM NaCl, 5 mM KCl, 1 mM MgCl_2_·6H_2_O, 2 mM CaCl_2_, 1 mM glucose, and 10 mM HEPES (pH 7.4). We then incubated them with 1 μM Fluor‐4‐AM for 40 min at 37℃. We then mounted the coverslips with the Fluo‐4‐AM‐loaded cells on a chamber positioned on the movable stage. We obtained images using a confocal microscope (Zeiss LSM 700) with a water‐immersion objective lens (40×). We used a 488 nm Argon laser to excite Fluo‐4‐AM, and measured emission signal using a bandpass filter (505–550 nm). We recorded the confocal images every 1.5 s for 460 s. The illumination intensity was limited to 0.5%–0.7% of the laser output. To measure the effect of CNO alone on intracellular Ca^2+^ levels in hM4Di‐expressing astrocytes, we recorded green fluorescence images every 0.5 s for 10 s (baseline) using a Lionheart FX Automated Microscope with a 10× phase lens. We then injected 10 μl of 10 μM CNO into the cells using the injector of the Lionheart FX Automate Microscope. We recorded images every 0.5 s for 170 s.

### 2.11 Statistical analysis

All data are presented as means ± SEM (in vivo data) or means ± SD (in vitro data), as indicated in the figure legends. A Student's *t*‐test was used to compare two experimental groups for RT‐PCR analysis. All other datasets were analyzed by one‐way ANOVA with Bonferroni's post hoc tests (in vitro experiments and passive avoidance test) using SPSS software (version 18.0; SPSS Inc.). Statistical significance was established at *p* < .05. The sample sizes for all experiments were chosen to ensure adequate statistical power on the basis of the G*power 3.1 software.^25^

## 3 RESULTS

### 3.1 Chemogenetic stimulation of G_i_ signaling in hippocampal astrocytes attenuates local inflammation

**FIGURE 1:**
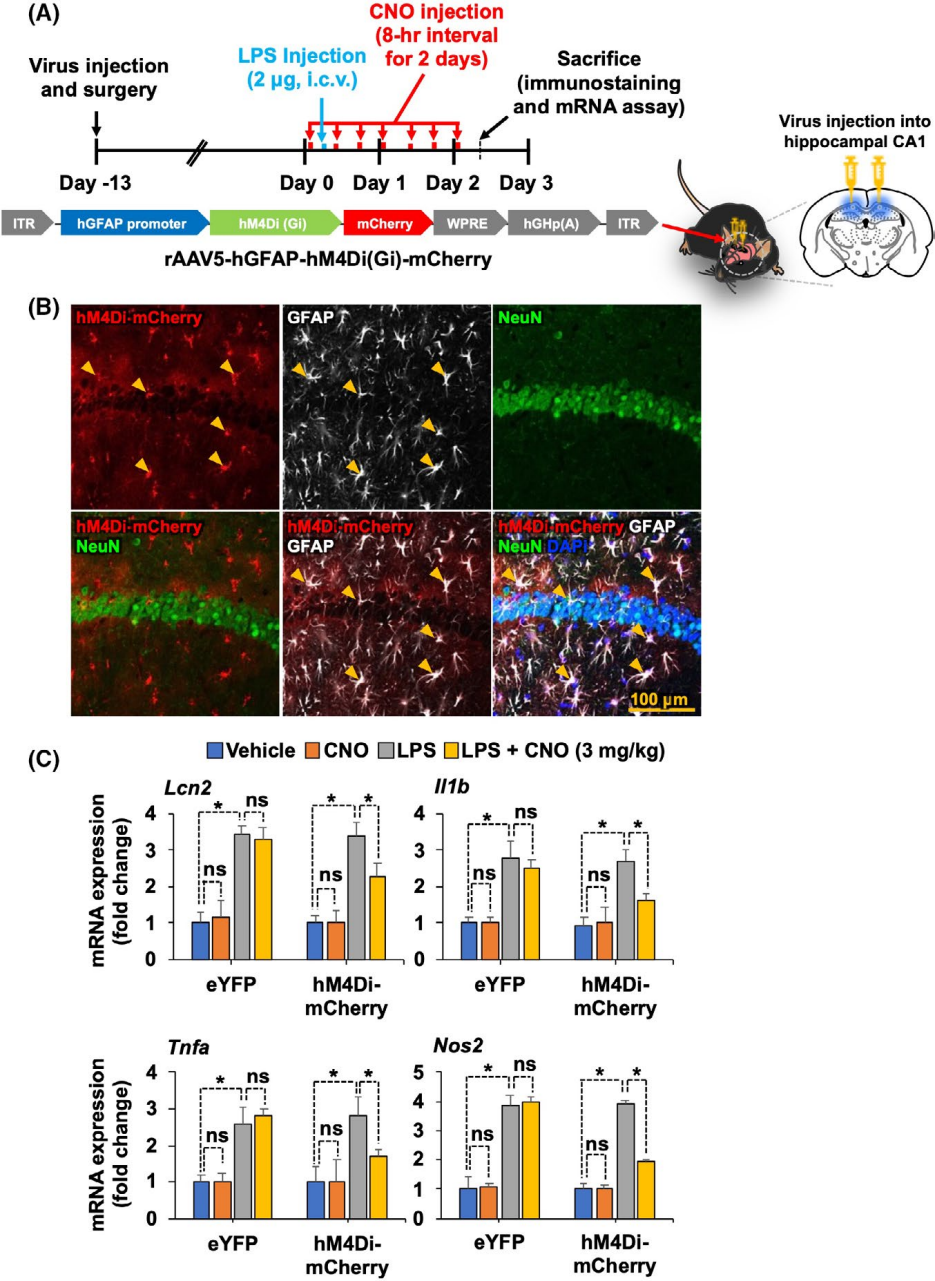
Selective expression and activation of astrocytic hM4Di inhibit LPS‐induced expression of proinflammatory mediators in the mouse hippocampus. (A) Schematic diagram showing the timeline of experimentation and AAV vector constructs. (B) Confocal images of mCherry labeling and immunofluorescence analysis. Astrocytes were immunohistochemically labeled with GFAP (white), and neurons were labeled with NeuN (green). Nuclei were stained with DAPI (blue). Arrowheads indicate the colocalization of hGFAP‐hM4Di‐mCherry and cell‐type‐specific markers. Scale bar, 200 μm. (C) After behavior tests, mice were sacrificed and total mRNA was extracted from the hippocampal tissues of each group. RT‐PCR was performed to assess the expression levels of *Lcn2*, *Il1b*, *Tnfa*, and *Nos2* mRNA expression profiles are displayed as the fold increase of gene expression normalized to *Gapdh* mRNA levels. Results are expressed as means ± SEM (*n* = 4). *p < .05 between the indicated groups (Student's *t*‐test). eYFP, AAV5‐hGFAP‐eYFP; hM4Di‐mCherry, AAV5‐hGFAP‐hM4Di‐mCherry; ns, not significant

**FIGURE 2:**
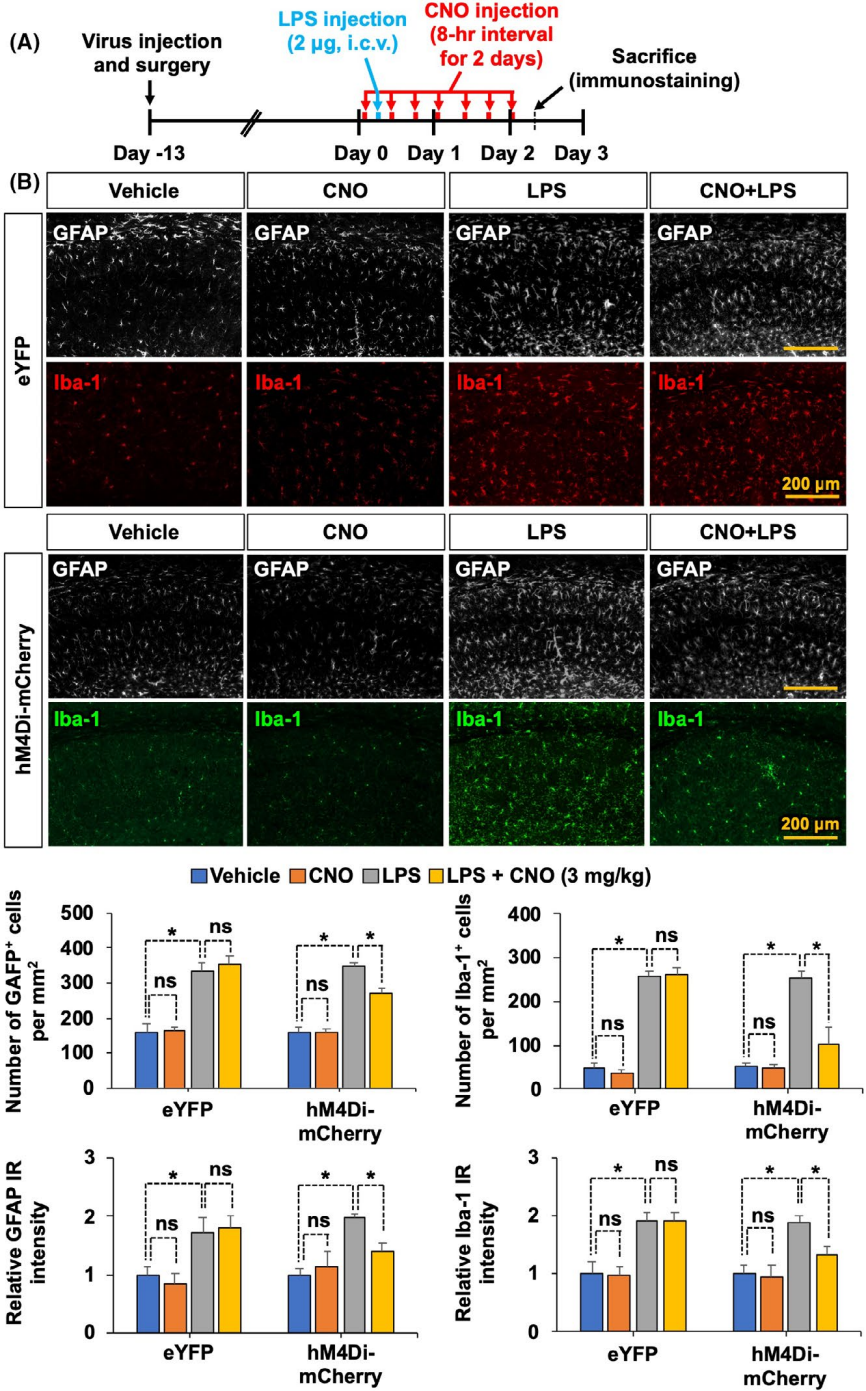
Selective activation of astrocytic hM4Di inhibits LPS‐induced glial activation in the mouse hippocampus. (A) Schematic diagram showing the timeline of experimentation. (B) Immunofluorescence staining and image analysis were performed. Astrocytes were immunohistochemically labeled with GFAP (white), and microglia were labeled with Iba‐1 (red or green). Quantitative analysis of GFAP‐positive astrocytes and Iba‐1‐positive microglia as well as relative GFAP and Iba‐1 immunoreactivity (IR) intensity in the hippocampus are presented in adjacent graphs. Scale bar, 200 μm. Results are expressed as means ± SEM (*n* = 4). *p < .05 between the indicated groups (one‐way ANOVA with Bonferroni's post hoc test). eYFP, AAV5‐hGFAP‐eYFP; hM4Di‐mCherry, AAV5‐hGFAP‐hM4Di; IR, immunoreactivity; ns, not significant

To investigate the role of astrocytic G_i_ signaling in neuroinflammation, the muscarinic receptor variant hM4Di fused to a red fluorescent protein mCherry was expressed in astrocytes within the hippocampal CA1 region. To achieve this, adeno‐associated virus incorporating a GFAP promoter (AAV5‐hGFAP‐hM4Di‐mCherry) was directly injected into the hippocampus. The G_i_‐coupled DREADD was then stimulated with CNO, delivered i.p. (Figure 1A). To confirm the astrocyte‐targeted expression of the hM4Di‐mCherry protein, we performed immunostaining with cell type‐specific antibodies in the hippocampal sections of injected mice, and found that the cells expressing hM4Di‐mCherry were mostly GFAP‐positive astrocytes, and not NeuN‐positive neurons (Figure 1B and Figure S1). To examine whether G_i_‐mediated activation of hippocampal astrocytes can alter local inflammation, we used a mouse model of LPS‐induced neuroinflammation. Using this model, we measured the mRNA expression of proinflammatory mediators, such as *Lcn2*, *Il1b*, *Tnfa*, and *Nos2*, in the hippocampus using real‐time PCR. Administration of CNO at 3 mg/kg (Figure 1C) but not at 1 mg/kg (Figure S2A,B) decreased the LPS‐induced levels of *Lcn2*, *Il1b*, *Tnfa*, and *Nos2* mRNA. Similarly, the role of astrocytic G_i_ signaling in neuroinflammation was further evaluated by the assessment of the state of glial activation in the hippocampus of LPS‐injected mice (Figure 2A). Brain sections were immunostained with anti‐GFAP and anti‐Iba‐1 antibodies to label the astrocytes and microglia, respectively (Figure 2B). Immunofluorescence analysis revealed that chemogenetic stimulation of G_i_ signaling via administration of CNO at 3 mg/kg diminished the LPS‐induced increase in the number of GFAP‐positive astrocytes and Iba‐1‐positive microglia, as well as the relative intensity of both GFAP and Iba‐1 immunoreactivity in the hippocampus. These findings suggest that astrocytic G_i_ pathway plays an inhibitory role in neuroinflammation.

### 3.2 Chemogenetic stimulation of G_i_ signaling in hippocampal astrocytes ameliorates LPS‐induced cognitive impairment in mice

**FIGURE 3:**
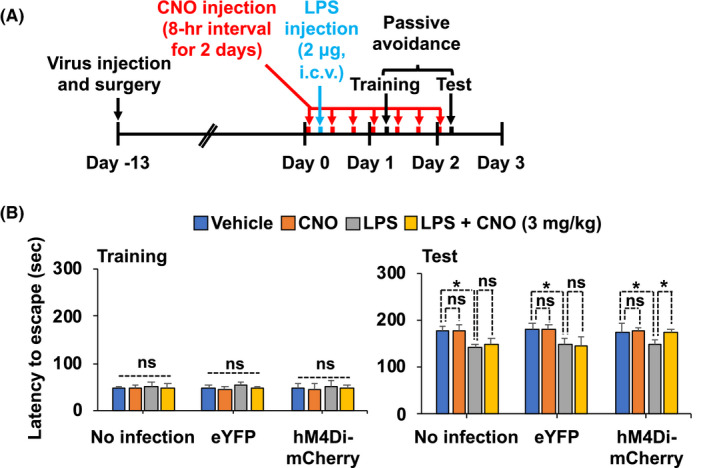
Selective activation of astrocytic hM4Di alleviates LPS‐induced cognitive impairment. (A) Schematic diagram showing the timeline of experimentation. (B) Cognitive impairment was evaluated using the passive avoidance test. The results are expressed as means ± SEM (*n* = 5 for each group). *p < .05 between the indicated groups (one‐way ANOVA with Bonferroni's post hoc test). eYFP, AAV5‐hGFAP‐eYFP; hM4Di‐mCherry, AAV5‐hGFAP‐hM4Di‐mCherry; ns, not significant

Accumulating evidence suggests that neuroinflammation is associated with impaired cognitive function in diverse neuropathological conditions.^26^, ^27^ To examine whether activation of astrocytic G_i_ signaling in the CA1 hippocampus could alleviate LPS‐induced cognitive impairment in mice, we performed the passive avoidance test. The hM4Di‐expressing mice received CNO (1 or 3 mg/kg, i.p.) seven times (8 h intervals) for 2 days (Figure 3A and Figure S2). The chemogenetic stimulation of astrocytic G_i_ signaling (CNO at 3 mg/kg) increased the latency to escape in LPS‐injected animals 24 h after a foot shock, indicating that astrocytic G_i_ signaling ameliorates LPS‐induced cognitive deficit (Figure 3B, *right*). The latency during the training trial did not differ among the experimental and control groups, indicating that all the mice had similar responses to the testing environment (Figure 3B, *left*). The escape latency was not affected by CNO treatment at a low dose (1 mg/kg) (Figure S2C). These results imply that strong activation of G_i_ signaling in hippocampal astrocytes has an inhibitory effect on LPS‐induced neuroinflammation and on subsequent cognitive impairment in mice.

### 3.3 Chemogenetic stimulation of G_i_ signaling in cultured astrocytes attenuates LPS‐induced nitric oxide production

**FIGURE 4:**
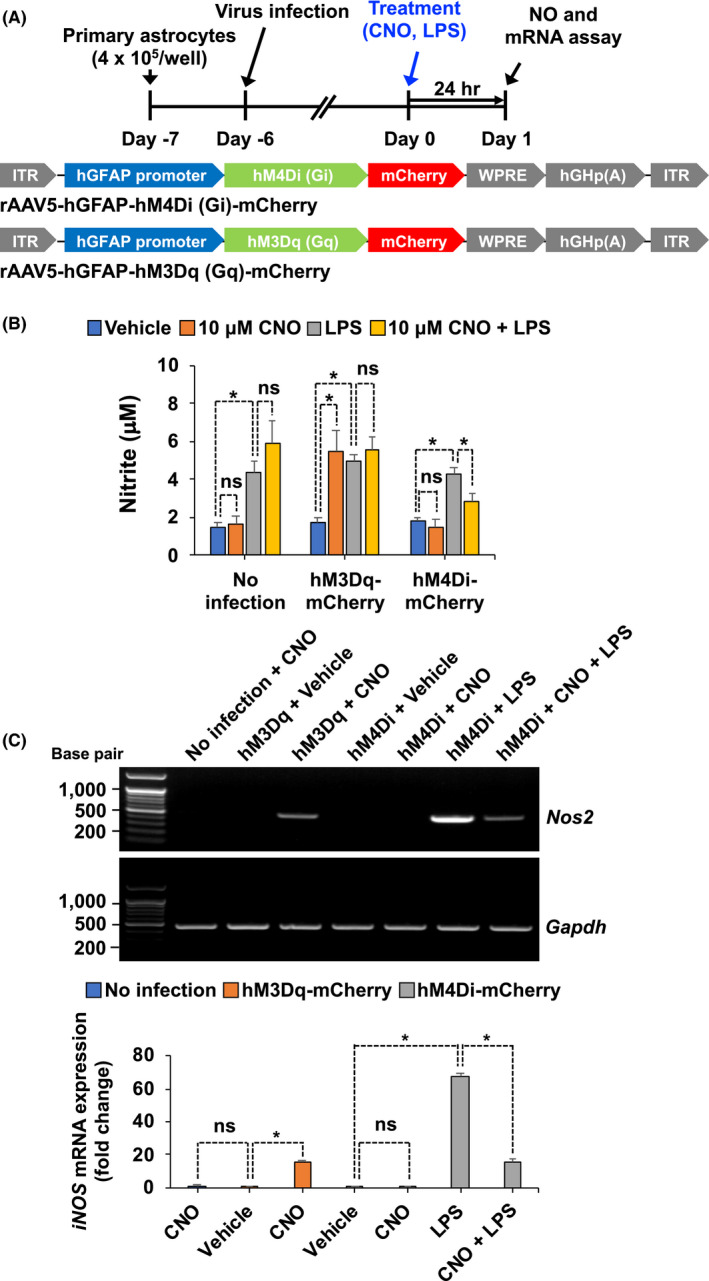
Stimulation of astrocytic G_i_ signaling in culture decreases LPS‐induced levels of nitrite and expression of *Nos2* mRNA. (A) Schematic diagram showing the timeline of experimentation and AAV vector constructs. (B) Primary astrocyte cultures were treated with CNO and LPS for 20 min. The levels of nitrite in culture media were measured after 24 h. (C) Total cellular RNA was extracted 24 h after treatment. Expression levels of *Nos2* mRNA were determined by RT‐PCR. Data were normalized to mRNA levels of *Gapdh*, and results are expressed as means ± SEM (*n* = 6). *p < .05 between the indicated groups (Student's *t*‐test). eYFP, AAV5‐hGFAP‐eYFP; hM3Dq‐mCherry, AAV5‐hM3Dq‐mCherry; hM4Di‐mCherry, AAV5‐hM4Di‐mCherry; ns, not significant

Since nitric oxide (NO) production has been used as an indicator of inflammation in astrocytes,^28^ we investigated the effect of astrocytic G_i_ signaling activation on LPS‐induced NO production. Primary astrocytes were infected with a AAV5‐hGFAP‐hM4Di‐mCherry virus construct and stimulated with LPS for 24 h (Figure 4A). Subsequently, the accumulated nitrite in the culture media was estimated using Griess reaction as an index for NO synthesis. Exposure of primary astrocyte cultures to LPS markedly increased the levels of nitrite in the culture media (Figure 4B). However, hM4Di activation by CNO treatment in primary astrocytes significantly decreased LPS‐induced NO production. We also used a positive control chemogenetic stimulation of the G_q_‐signaling pathway, by infecting astrocyte cultures with a AAV5‐hGFAP‐hM3Dq‐mCherry virus construct. As expected, the activation of the astrocytic G_q_‐signaling pathway using CNO increased the levels of nitrite in the media. Next, to investigate whether the inhibitory effect of astrocytic G_i_ signaling on LPS‐induced NO production was mediated by iNOS suppression, we performed RT‐PCR analysis and found that activation of G_i_ significantly decreased LPS‐induced expression of *Nos2* mRNA (Figure 4C). These findings support the inhibitory role of astrocytic G_i_ signaling in neuroinflammation.

### 3.4 Chemogenetic stimulation of G_i_ signaling in cultured astrocytes attenuates LPS‐induced intracellular Ca^2+^ transients

**FIGURE 5:**
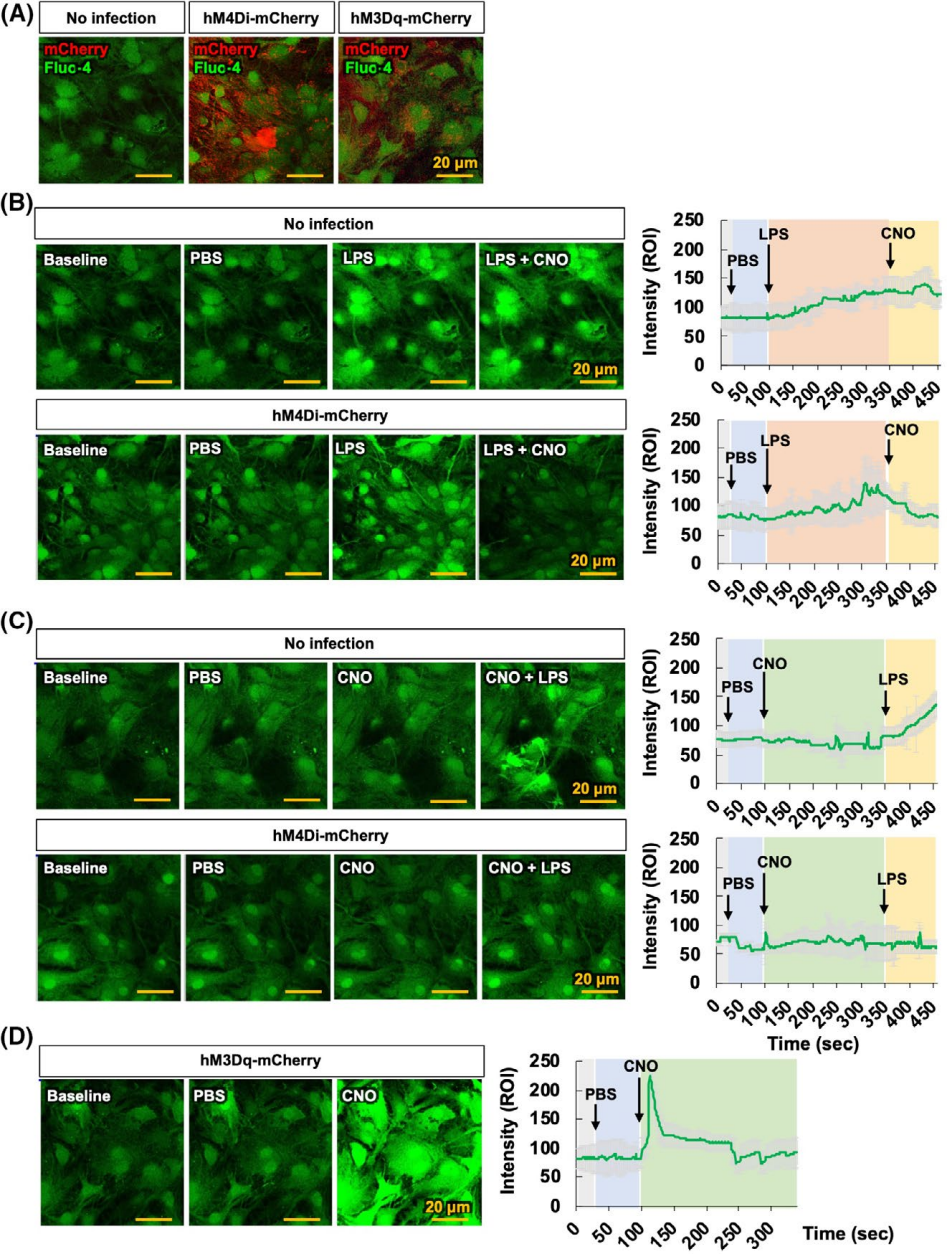
G_i_ signaling activation in astrocytes inhibits LPS‐induced intracellular Ca^2+^ levels. (A) Representative images showing co‐localization of Fluo‐4‐AM (green) and mCherry (red) in negative controls, hM4Di‐mCherry‐, and hM3Dq‐mCherry‐infected primary astrocyte cultures. (B, C) Representative images showing Ca^2+^ transients after treatment with CNO (10 μM) in negative controls and hM4Di‐expressing primary astrocytes loaded with Fluo‐4‐AM. Astrocytes were treated with PBS at 20 s, LPS (100 ng/ml) at 100 s, and CNO at 350 s (B); or treated with PBS at 20 s, CNO at 100 s, and LPS (100 ng/ml) at 350 s (C). Representative traces show the change of intracellular Ca^2+^ in astrocytes as induced by LPS in the absence or presence of CNO. (D) hM3Dq‐expressing primary astrocytes loaded with Fluo‐4‐AM exhibited Ca^2+^ transients after treatment with CNO. Astrocytes were treated with PBS at 20 s and CNO at 100 s. Representative traces show the change of intracellular Ca^2+^ in astrocytes induced by CNO. Arrows indicate the treatment time for PBS, LPS, and CNO in the traces. Results are expressed as means ± SEM (*n* = 6). Scale bar, 20 μm. eYFP, AAV5‐hGFAP‐eYFP; hM3Dq‐mCherry, AAV5‐hM3Dq‐mCherry; hM4Di‐mCherry, AAV5‐hM4Di‐mCherry

Ca^2+^ signals in astrocytes change both acutely and chronically in response to brain insults, such as injury and inflammation.^29^ However, it is unclear how G_i_ signaling in astrocytes affects Ca^2+^ signals during neuroinflammation. To test this, we performed Ca^2+^ imaging in cultured astrocytes expressing either hM4Di, hM3Dq (a positive control), or without infection (a negative control), in which cultured astrocytes were loaded with Fluo‐4‐AM as shown in Figure 5A. The primary astrocytes expressing hM4Di were imaged before and after application of PBS, LPS, and CNO (10 μM) (Figure 5B,C). The data revealed that CNO application following LPS treatment reduced LPS‐induced intracellular Ca^2+^ levels in hM4Di‐expressing astrocytes (Figure 5B). The application of CNO prior to LPS also prevented LPS‐induced upregulation of intracellular Ca^2+^ levels in hM4Di‐expressing astrocytes (Figure 5C). However, hM3Dq‐expressing astrocytes showed an increase in intracellular Ca^2+^ levels following CNO application (Figure 5D). As shown in Figure 5C, CNO application acutely increased intracellular Ca^2+^ levels in hM4Di‐expressing astrocytes. Thus, to better characterize the effect of intracellular Ca^2+^ levels in hM4Di‐expressing astrocytes, we measured intracellular Ca^2+^ levels in hM4Di‐expressing astrocytes treated with CNO alone. Treatment with CNO alone increased intracellular Ca^2+^ levels in hM4Di‐expressing astrocytes (Figure S3). Similarly, chemogenetic activation of either the G_q_ or G_i_ pathway increased intracellular Ca^2+^ in astrocytes.^9^, ^30^, ^31^, ^32^ However, whereas G_q_ pathway activation resulted in a long‐lasting increase of Ca^2+^ activity (Figure 5D), the G_i_ pathway‐induced intracellular Ca^2+^ transient wanes in time (Figure S3). Collectively, these findings suggest that the alteration in intracellular Ca^2+^ levels following G_i_ or G_q_ pathway activation in astrocytes may reflect the features of reactive astrocytes associated with neuroinflammation.

## 4 DISCUSSION

Our findings demonstrate that chemogenetic stimulation of the astrocytic G_i_ signaling pathway in the hippocampus plays an inhibitory role in neuroinflammation and subsequent cognitive decline in mice. Stimulation of G_i_ signaling was sufficient to inhibit proinflammatory activation of astrocytes, which correlated with intracellular Ca^2+^ transients.

In this study, we have shown that long‐term hM4Di activation ameliorates LPS‐induced production of proinflammatory mediators and cognitive impairment, suggesting that G_i_ signaling could lead to suppression of inflammatory activation of astrocytes and concurrent neuroinflammatory changes. The role of astrocytic GPCR signaling in neuroinflammation has been previously reported.^33^ The astrocyte dopamine receptor (DRD)‐2 coupled to G_i_ ^34^, ^35^ has been found to decrease in the aging brain,^36^ implying a potential involvement of DRD2 in aging‐related neuroinflammation. It has been reported that DRD2‐deficient astrocytes produce higher levels of proinflammatory molecules.^37^ This effect is mediated through inhibition of αB‐crystallin signaling, a small heat‐shock protein known to negatively regulate the production of proinflammatory mediators and to exhibit neuroprotective effects. Intriguingly, DRD2‐deficient astrocytes also display robust upregulation of GFAP expression with a reactive morphology in the substantia nigra and the striatum of aged mice,^33^ suggesting a possible link between astrocytic G_i_ signaling and age‐related neuroinflammation and subsequent behavioral impairment. Conversely, dopaminergic signaling triggered by the stimulation of G_i_‐coupled DRD3 promotes a proinflammatory phenotype in astrocytes.^38^ Moreover, G_i_‐coupled P2Y12R and P2Y14R signaling has also been reported to be involved in proinflammatory activation of astrocytes and immune cells.^39^, ^40^, ^41^, ^42^, ^43^, ^44^, ^45^

In this study, chemogenetic stimulation of G_i_ signaling in primary astrocytes led to the downregulation of iNOS and subsequent NO production. Elevated levels of NO produced within the CNS are associated with the pathogenesis of neuroinflammatory and neurodegenerative diseases.^46^ Astrocytes express iNOS and produce high levels of NO in response to a wide range of stimuli, including pathogens, which have been implicated in neuroinflammatory processes.^47^ Several transcription factors are involved in transactivation of the *Nos2* gene, such as nuclear factor‐κB (NF‐κB) and activator protein‐1 (AP‐1), as well as various members of the CCAAT/enhancer‐binding protein (CEBP), activating transcription factor (ATF)/cAMP response element‐binding protein (CREB), and signal transducer and activator of transcription (STAT) families of transcriptional factors.^48^ In addition, the second messenger cAMP has been implicated in *Nos2* gene regulation as well. It has been reported that the increase of intracellular cAMP levels inhibits LPS‐stimulated iNOS or proinflammatory cytokine expression in several cell types.^49^ Paradoxically, astrocytic iNOS expression and NO production have been reported to be both enhanced ^50^, ^51^ and suppressed ^52^, ^53^ by cAMP; however, studies using chemogenetic approaches demonstrate that activation of G_i_ signaling in astrocytes suppresses cAMP.^54^ As our study revealed an inhibitory effect of the astrocytic G_i_ pathway on LPS‐induced NO production, further studies are necessary to elucidate the precise signaling pathways downstream of G_i_, including those involving cAMP and potassium channels,^55^ in astrocytes.

Our study demonstrates that chemogenetic stimulation of the astrocytic G_i_ pathway attenuated LPS‐induced intracellular Ca^2+^ levels, supporting an inhibitory role of G_i_ signaling in the inflammatory activation of astrocytes. Astrocytes are not electrically excitable cells, and their responses to external input are best represented by the elevation of intracellular Ca^2+^ levels.^56^ Multiple sources contribute to astrocytic Ca^2+^ elevation, among which the endoplasmic reticulum (ER) is known to be the major source of Ca^2+^, the release of which is triggered via activated inositol triphosphate (IP_3_) receptors.^57^ Reactive astrocytes show elevated Ca^2+^ levels upon various inflammatory stimuli.^58^ It has been reported that the upregulation of astrocytic Ca^2+^ is also essential for GFAP upregulation (a marker for reactive astrocytes) in diverse neuropathologies, including AD,^59^ Alexander disease,^60^ photothrombosis,^59^ and traumatic brain injury,^61^ whereas abrogation of aberrant Ca^2+^ signals (via IP_3_ receptor KO, etc.) strongly suppresses GFAP upregulation and subsequent inflammatory phenotypes.

Astrocytes express toll‐like receptor type 4 (TLR4), which belongs to the TLR family in the vertebrate immune system and specifically recognizes LPS.^62^ On astrocytes, LPS decreases the expression of proteins such as gap junction proteins ^63^ and increases the expression of others such as GFAP, s100β, IL‐1, and TNF‐α.^64^, ^65^, ^66^, ^67^ Treating primary astrocytes with LPS stimulates the expression and activity of L‐type voltage‐operated calcium channels (VOCCs), which induces Ca^2+^ signals.^68^ Functional L‐type VOCCs are important for the activation of astrocytes in response to LPS, and L‐type VOCC blockers cancel these effects. Knocking down/out the L‐type VOCC subunit Cav1.2 in astrocytes further confirmed these results. Another study demonstrated that LPS stimulation also affects the astrocytes’ intracellular Ca^2+^ signaling. LPS‐elicited Ca^2+^ transients occurred in a concentration‐dependent bell‐shaped distribution. Besides, the TLR4 antagonist Rhodobacter sphaeroides LPS (LPS‐RS) blocked the Ca^2+^‐induced peak.^69^ The authors have found that LPS‐induced Ca^2+^ transients oscillate, Na^+^/K^+^‐ATPase is downregulated, and the actin filaments are disorganized.^69^ The Na^+^/K^+^‐ATPase is an energy‐transducing pump, and its expression decreased with time. Modulating this pump's activity affects intracellular Na^+^ concentration, which in turn changes intracellular Ca^2+^ concentration via Na^+^‐Ca^2+^ exchanges.^70^ Therefore, these data implicate L‐type Ca^2+^ channels in the LPS‐induced activation of astrocytes.^71^ However, identifying the molecular pathways involved in L‐type VOCCs modulation by LPS requires further studies.

In conclusion, our findings suggest that the astrocytic G_i_ pathway plays an inhibitory role in neuroinflammation via downregulation of proinflammatory mediators, NO production, and intracellular Ca^2+^ levels. These results provide opportunities to identify potential cellular and molecular targets for the control of neuroinflammation.

## DISCLOSURE

The authors declare no conflicts of interest in regards to this manuscript.

## ETHICS APPROVAL STATEMENT

All animal experiments were performed according to approved animal protocols and guidelines established by the Animal Care Committee of Kyungpook National University (No. KNU 2019‐09).

## AUTHOR CONTRIBUTIONS

J.‐H. K. performed experiments and analyzed data; J.‐H. K. W.H. L., and K. S. designed the study; J.‐H. K., M. H. R., and K. S. wrote the paper. All authors critically reviewed the text and figures.

## DATA AVAILABILITY STATEMENT

All data pertinent to this work are contained within this manuscript or available upon request. For requests, please contact: Kyoungho Suk, Kyungpook National University, ksuk@knu.ac.kr.

## Supporting information

Figure S1

Figure S2

Figure S3
