# Supplementary material for: Chemogenetic stimulation of the Gi pathway in astrocytes suppresses neuroinflammation
Source: Pharmacol Res Perspect. 2021 Oct 22;9(6):e00822. doi: 10.1002/prp2.822 (PMC8532135; doi:10.1002/prp2.822)
Supplement: Supplementary file 1 — Figure S1 [file PRP2-9-e00822-s001.pdf]

Supplementary Figure S1

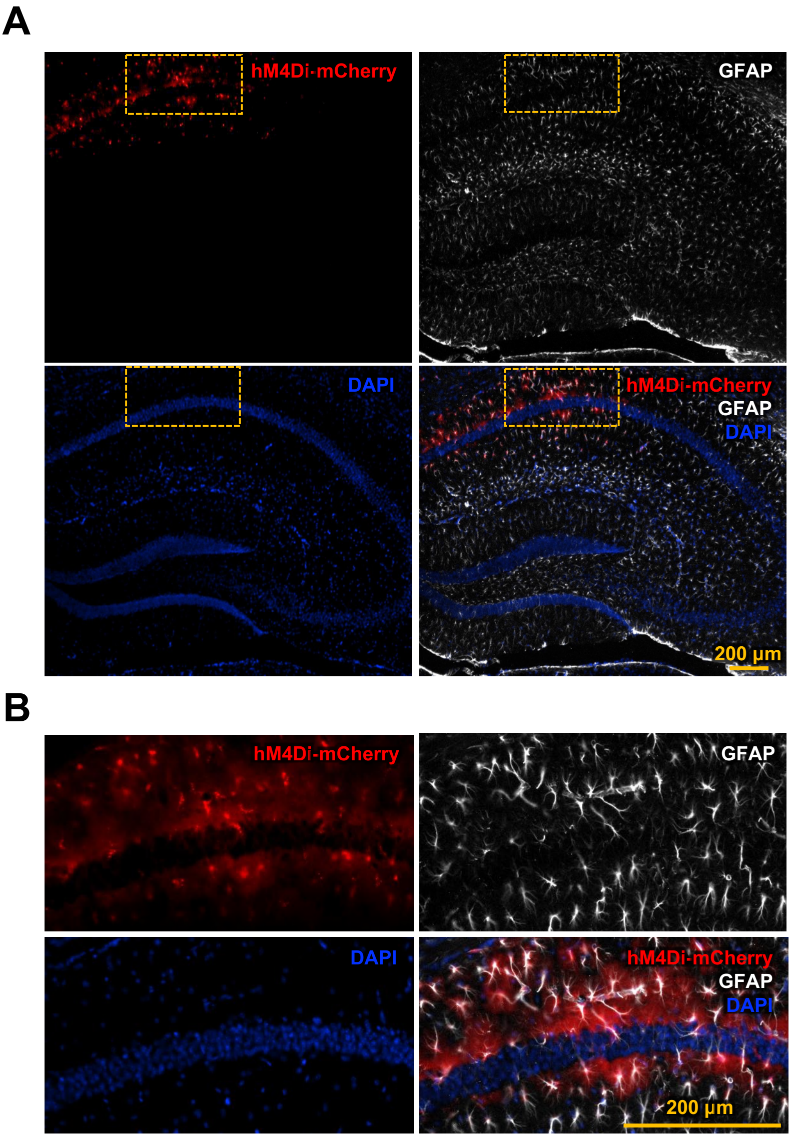

Supplementary Figure S1. Selective expression of hM4Di-mCherry in the hippocampal astrocytes. Representative low magnification images showing virus-induced hM4Di-mCherry expression (red), and immunostaining for GFAP (white), and DAPI (blue) in the hippocampal CA1 region (**A**). The dotted box (yellow) shows the hippocampal CA1 area magnified in Figure 1B (**B**).
