# Supplementary material for: Chemogenetic stimulation of the Gi pathway in astrocytes suppresses neuroinflammation
Source: Pharmacol Res Perspect. 2021 Oct 22;9(6):e00822. doi: 10.1002/prp2.822 (PMC8532135; doi:10.1002/prp2.822)
Supplement: Supplementary file 2 — Figure S2 [file PRP2-9-e00822-s003.pdf]

Supplementary Figure S2

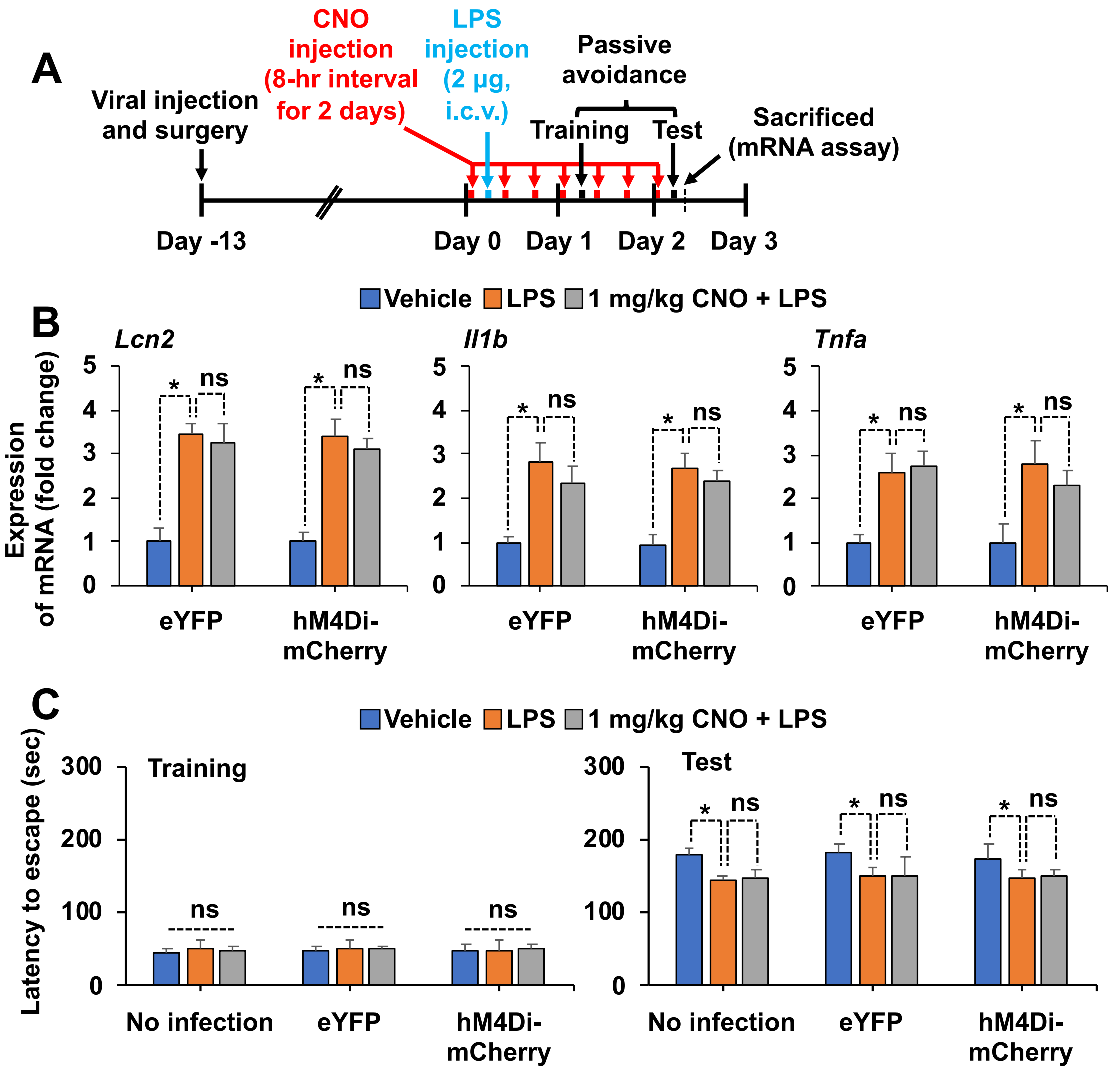

Supplementary Figure S2. A low dose of CNO does not alter LPS-induced neuroinflammation or cognitive impairments in hM4Di-expressing mice. (**A**)Schematic diagram showing the timeline of experimentation. (**B**) Total mRNA was extracted from the hippocampal tissues of each treatment group, and RT-PCR was performed to assess the expression levels of *Lcn2*, *Il1b*, and *Tnfa* mRNA. The graph displays quantitative results normalized to *Gapdh* mRNA levels. Results are expressed as means ± SEM ($n = 4$). *$p < 0.05$ between the indicated groups (Student's *t*-test). (**C**) Cognitive impairment was evaluated using the passive avoidance test. Results are expressed as means ± SEM ($n = 4$). *$p < 0.05$ between the indicated groups (one-way ANOVA with Bonferroni's post hoc test). hM4Di-mCherry, AAV5-hGFAP-hM4Di-mCherry; eYFP, AAV5-hGFAP-eYFP; ns, not significant.
