# Supplementary material for: Chemogenetic stimulation of the Gi pathway in astrocytes suppresses neuroinflammation
Source: Pharmacol Res Perspect. 2021 Oct 22;9(6):e00822. doi: 10.1002/prp2.822 (PMC8532135; doi:10.1002/prp2.822)
Supplement: Supplementary file 3 — Figure S3 [file PRP2-9-e00822-s002.pdf]

Supplementary Figure S3

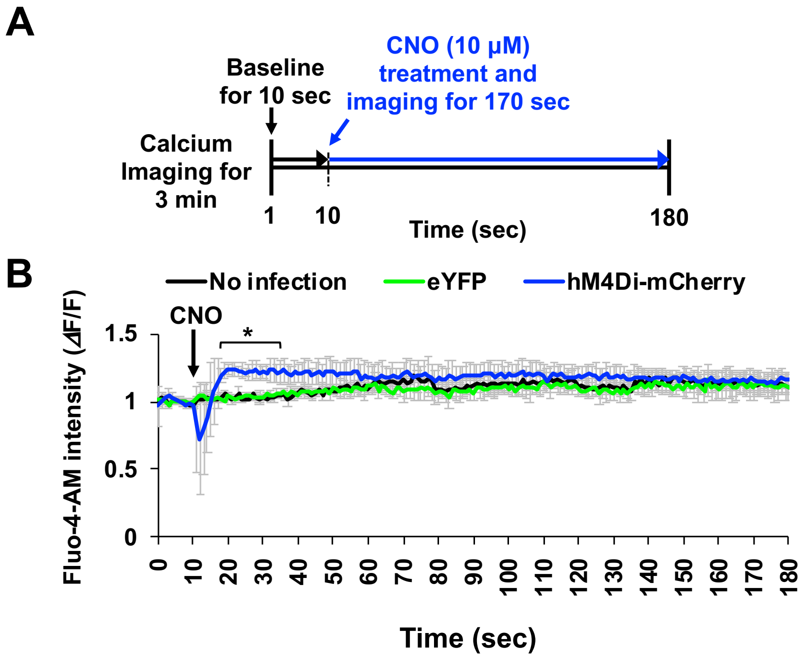

Supplementary Figure S3. Chemogenetic stimulation of cultured astrocytes increases intracellular Ca^{2+} levels. A, Experimental timeline. The hM4Di-expressing primary astrocytes loaded with Fluo-4-AM exhibited Ca^{2+} transients after treatment with CNO (10 μM). CNO was added at 10 s. **B**, Fluo-4-AM fluorescence traces $(F\text{-}F_0)/F_0$ from primary astrocytes (180 s). The "no infection" and eYFP groups had unaltered Ca^{2+} levels. Results are expressed as the mean ± SD (n = 5). $*p < 0.05$ between the indicated groups (one-way ANOVA).
